# Cognition and behaviour in frontotemporal dementia with and without amyotrophic lateral sclerosis

**DOI:** 10.1136/jnnp-2020-323969

**Published:** 2020-10-14

**Authors:** Jennifer A Saxon, Jennifer C Thompson, Jennifer M Harris, Anna M Richardson, Tobias Langheinrich, Sara Rollinson, Stuart Pickering-Brown, Amina Chaouch, John Ealing, Hisham Hamdalla, Carolyn A Young, Dan Blackburn, Tahir Majeed, Claire Gall, Matthew Jones, Julie S Snowden

**Affiliations:** 1 Cerebral Function Unit, Manchester Centre for Clinical Neurosciences, Salford Royal NHS Foundation Trust, Salford, UK; 2 Division of Neuroscience and Experimental Psychology, The University of Manchester, Manchester, UK; 3 Motor Neurone Disease Care Centre, Manchester Centre for Clinical Neurosciences, Salford Royal NHS Foundation Trust, Salford, UK; 4 Department of Neurology, The Walton Centre NHS Foundation Trust, Liverpool, UK; 5 Institute of Systems, Molecular and Integrative Biology, University of Liverpool, Liverpool, UK; 6 Sheffield Institute for Translational Neuroscience (SITraN), The University of Sheffield, Sheffield, UK; 7 Neurology, Lancashire Teaching Hospitals NHS Foundation Trust, Preston, UK

## Abstract

**Objective:**

The precise relationship between frontotemporal dementia (FTD) and amyotrophic lateral sclerosis (ALS) is incompletely understood. The association has been described as a continuum, yet data suggest that this may be an oversimplification. Direct comparisons between patients who have behavioural variant FTD (bvFTD) with and without ALS are rare. This prospective comparative study aimed to determine whether there are phenotypic differences in cognition and behaviour between patients with FTD-ALS and bvFTD alone.

**Methods:**

Patients with bvFTD or FTD-ALS and healthy controls underwent neuropsychological testing, focusing on language, executive functions and social cognition. Behavioural change was measured through caregiver interview. Blood samples were screened for known FTD genes.

**Results:**

23 bvFTD, 20 FTD-ALS and 30 controls participated. On cognitive tests, highly significant differences were elicited between patients and controls, confirming the tests’ sensitivities to FTD. bvFTD and FTD-ALS groups performed similarly, although with slightly greater difficulty in patients with ALS-FTD on category fluency and a sentence-ordering task that assesses grammar production. Patients with bvFTD demonstrated more widespread behavioural change, with more frequent disinhibition, impulsivity, loss of empathy and repetitive behaviours. Behaviour in FTD-ALS was dominated by apathy. The *C9ORF72* repeat expansion was associated with poorer performance on language-related tasks.

**Conclusions:**

Differences were elicited in cognition and behaviour between bvFTD and FTD-ALS, and patients carrying the *C9ORF72* repeat expansion. The findings, which raise the possibility of phenotypic variation between bvFTD and FTD-ALS, have clinical implications for early detection of FTD-ALS and theoretical implications for the nature of the relationship between FTD and ALS.

## Introduction

An association between frontotemporal dementia (FTD) and amyotrophic lateral sclerosis (ALS) is well established on clinical, pathological and genetic grounds, yet the precise nature of the relationship remains controversial.

Up to 15% of people with ALS develop FTD^1^ and a similar proportion of people with FTD develop ALS.^2 3^ Similarities in profile of cognitive impairment have been identified in the two disorders, although more severe in FTD.^4^ TDP-43 pathology occurs in both conditions.^5^ So too do expansions in the *C9ORF72* gene.^6–9^ Such convergent evidence supports the notion of a spectrum or continuum of disease.^3 10^

On the other hand, some authors^11^ have reported distinct cognitive profiles in ALS and ALS-FTD, and explicitly argue against the notion of a continuum. Moreover, while FTD-ALS is pathologically homogenous, invariably being associated with TDP-43 pathology,^5^ half of FTD cases without ALS have alternative pathologies: tau or fused-in-sarcoma.^12^ Furthermore, of the three main genes implicated in FTD: *C9ORF72*, *PGRN* and *MAPT*, only *C9ORF72* is associated with FTD-ALS. Therefore FTD-ALS is predictive of a pathological and genetic signature in a way that FTD alone is not. It would be reasonable to infer that not all patients with FTD are equally vulnerable to developing ALS.

An important question is whether it is possible to identify potentially vulnerable patients with FTD on clinical grounds. Specifically, are there phenotypic differences between patients who have FTD with and without accompanying ALS? The issue has clinical relevance for early detection of FTD-ALS and patient management as well as having theoretical implications for the relationship between FTD and ALS.

There is some limited evidence for phenotypic differences between FTD and FTD-ALS. FTD encompasses three canonical clinical syndromes: behavioural variant FTD (bvFTD), semantic dementia (SD), also known as semantic variant primary progressive aphasia and progressive non-fluent aphasia (PNFA)/non-fluent variant primary progressive aphasia. SD and PNFA, at least in their pure forms, are rarely associated with FTD-ALS,^2 13^ raising the possibility of a more uniform clinical phenotype in FTD-ALS, associated with behaviour change, compared with FTD alone.

There have, however, been few direct comparisons of cognition and behaviour in bvFTD and FTD-ALS and the limited evidence is inconsistent. De Silva *et al* 14 reported greater behavioural change in bvFTD than FTD-ALS whereas Lillo *et al* 15 found no differences in frequency of behavioural symptoms, although identified higher rates of aphasia and psychosis in FTD-ALS. Our own retrospective study of bvFTD and FTD-ALS raised the possibility of more frequent agrammatism and impaired syntactic comprehension in FTD-ALS and greater social disinhibition and reduced empathy in bvFTD.^16^ That study was limited by its retrospective nature, reliance on presence/absence of symptoms or deficits rather than quantitative measurement, and lack of control for motor deficits in FTD-ALS. In that and other studies there was no exploration of the potential genetic contribution to clinical phenotype.

The aim of the present study was to compare cognition and behaviour in bvFTD and FTD-ALS. The study incorporates assessment of language, executive functions and social cognition, inclusion of appropriate motor controls, behavioural and neuropsychiatric measures applicable to FTD, and analysis of genetic contributions to the cognitive and neuropsychiatric profiles. We anticipated greater behavioural change in bvFTD than FTD-ALS, with changes in FTD-ALS being dominated by apathy. We also predicted that deficits in language processing would occur more frequently in FTD-ALS. Given the known heterogeneity of FTD however, it was anticipated that there would be a degree of variation within and overlap between the groups, in part influenced by genetic contributions.

## Methods

This is a prospective cross-sectional comparative group study. It involved consecutive patients who agreed to participate and fulfilled the criteria for the study during the recruitment period.

### Participants

The study included patients with a clinical diagnosis of bvFTD or FTD-ALS, and healthy volunteers. Patients were recruited between December 2014 and September 2017 from specialist cognitive or motor neuron disease clinics at Salford Royal NHS Foundation Trust (the Cerebral Function Unit), the Walton Centre NHS Foundation Trust, Lancashire Teaching Hospitals NHS Trust and Sheffield Teaching Hospitals. Clinical diagnoses were made by specialist neurologists and supported in most cases by detailed neuropsychological evaluation. Patients fell into the mild to moderate range of impairment as measured by the Clinical Dementia Rating (CDR) scale, modified for use with patients who have FTD.^17^ All patients fulfilled contemporary diagnostic criteria for bvFTD.^18^ Patients with FTD-ALS also met El Escorial criteria for ALS.^19^ Patients with FTD-ALS were excluded if they fell into the ‘very severe’ range of disability (score <12), as measured by the ALS Functional Rating Scale revised,^20^ or if they required mechanical respiratory support. Healthy controls were recruited through the Cerebral Function Unit’s ethically approved research register (Salford Royal NHS Foundation Trust) or Join Dementia Research. Participants were excluded if there was evidence of significant cerebrovascular disease, history of head injury, alcohol or drug abuse, or other neurological or medical disorders that might affect cognition. Participants were required to have premorbid fluency in English, as several tasks were designed to assess language. Patients’ caregivers were invited to complete behavioural interviews/questionnairesAssessments were carried out in a hospital setting or in the patient’s home according to personal preference.

### Cognitive assessment

Assessment of participants focused on language, executive skills and social cognition, known to be impaired in FTD and ALS. Language tests included The Graded Naming test^21^ of confrontation naming, the Object and Action naming test^22^ allowing comparison of noun and verb naming, the Pyramids and Palm Trees test^23^ of semantic association for words and pictures, the Psycholinguistic Assessment of Language Processing in Aphasia (PALPA)^24^ test of spelling to dictation (subtest 40) and sentence comprehension (subtest 55) and a locally developed sentence ordering test. The latter requires patients to rearrange five randomly presented printed words to form a sentence (eg, they went to the beach) and was included because of its proven sensitivity to grammatical impairments in FTD.^25^ Executive tests comprised letter and category fluency, and sorting tests from the Delis-Kaplan Executive Function System battery (DKEFS),^26^ and the Hayling and Brixton tests^27^ to assess response inhibition and rule abstraction and set shifting. Social cognition was assessed by a Judgement of Preference from Eye Gaze task^28^ and emotion recognition using the Ekman and Friesen faces.^29^ Assessment lasted 2–3 hours and was administered over separate sessions to patients to reduce fatigue. To accommodate patients’ motor difficulties either oral or written responses were permitted. For Verbal Fluency, a Verbal Fluency Index, which represents the average ‘thinking time’ per word, was calculated, as previously described.^30^

### Behaviour assessment

Behaviour assessment, of patients only, was carried out through caregiver interview. It included the neuropsychiatric inventory (NPI),^31^ which covers 12 behavioural dimensions, rated for both severity and frequency, and The Family Rating version of the Frontal Systems Behaviour Scale (FrSBe),^32^ a 46-item rating scale, yielding three subscale scores: apathy, disinhibition and executive dysfunction. The presence or absence of behavioural features (disinhibition, apathy/inertia, social/emotional change, stereotypies, dietary change) from the international consensus criteria for bvFTD^18^ was recorded through structured interview.

### Genetic analysis

Patients were invited to provide a blood sample to be screened for known FTD genes. Genotyping was carried out using the Ion PGM System for next generation sequencing. Testing for the hexanucleotide repeat expansion in *C9ORF72* was carried out using a repeat primed PCR method.^7^ Where patients were not screened this was for logistical reasons.

### Statistical analysis

Data were analysed using IBM SPSS Statistics V.25. Group comparisons were carried out using analysis of variance with post-hoc Gabriel tests or t-tests for demographic data and Kruskal-Wallis and Mann-Whitney tests for cognitive and behavioural data for which data were not normally distributed. Wilcoxon tests were used for related samples. χ^2^ and Fisher’s exact tests were used for categorical variables as appropriate. Significance values are shown in the tables uncorrected for multiple comparisons, to minimise the risk of masking potentially informative data: the relatively small sample size of the patient groups limits the power to detect significant differences between bvFTD and FTD-ALS. Corrected results are noted in the text.

## Results

### Study cohort

Seventy-one patients were approached and 46 agreed to participate (25 bvFTD and 21 FTD-ALS). Three patients (two bvFTD and one FTD-ALS) were later excluded due to diagnostic uncertainty. Forty healthy controls were initially recruited; however, to reduce age disparity between groups the ten youngest were excluded. It was not possible to collect behavioural data from four caregivers. The final cohort consisted of 23 patients with bvFTD, 20 patients with FTD-ALS and 30 healthy controls, together with 39 caregivers. 19/23 (82%) bvFTD and 11/20 (55%) patients with FTD-ALS fulfilled criteria for probable, as opposed to possible, bvFTD^18^: they had evidence both of functional decline and frontal and/or temporal atrophy on neuroimaging in addition to their cognitive and behavioural disorder. The group difference reaches statistical significance (χ^2^=3.9, p=0.05). Scans in two patients with bvFTD and six patients with FTD-ALS were reported to show generalised atrophy and one patient with bvFTD and one patient with FTD-ALS had a normal scan. Imaging was not available for one patients with bvFTD and two patients with FTD-ALS. Notably, two patients with bvFTD and two patients with FTD-ALS with generalised atrophy or a normal scan had a positive C9orf72 repeat expansion (see the Genetics section), providing confirmation of the FTD diagnosis. Of the FTD-ALS group, 13 had presented initially to an ALS clinic and 7 to a specialist dementia clinic. Eleven caregivers reported noticing cognitive symptoms first, six motor symptoms and three cognitive and motor symptoms simultaneously. Thirteen patients with FTD-ALS had some degree of bulbar involvement at the time of testing.

There were some demographic differences between the three groups (table 1). Post-hoc comparisons between group pairs showed that the control group included more female participants than both patient groups (p=0.03), controls were younger than the FTD-ALS group (p=0.03) and had more years of education than the bvFTD group (p=0.02). The bvFTD group had more years of illness than the FTD-ALS group. Other comparisons were non-significant.

**Table 1:** Participant demographics

	Control	bvFTD	FTD-ALS	ANOVA, t-test or χ^2^	P value
Number	30	23	20		
Sex male:female	9:21	14:9	12:8	χ^2^=6.6	0.04
Age at test mean (SD)	59 (8)	60 (7)	65 (8)	F(2,72)=3.32	0.03
Years of education mean (SD)	14 (3)	12 (3)	13 (3)	F(2,72)=3.86	0.03
Duration of symptoms Mean years (SD)	n/a	5 (4)	3 (1)	t=2.6	0.02
Disease severity* mean (SD)	n/a	9.3 (2.8)	9.5 (2.8)	t=−0.26	0.80

*Modified Clinical Dementia Rating scale,^17^ mean sum of boxes.

ANOVA, analysis of variance; bvFTD, behavioural variant FTD; FTD, frontotemporal dementia.

### Cognition

Kruskal-Wallis tests showed highly significant group differences on all cognitive tests, with p values of p<0.001 for all measures apart from the PALPA sentence comprehension and Brixton tests, which elicited significance levels of p=0.002. Subsequent Mann-Whitney U tests revealed that these striking differences lay between patients and controls (table 2). Only subtle differences were elicited between bvFTD and FTD-ALS. Patients with FTD-ALS performed more poorly on category fluency and showed a trend towards greater difficulty on sentence ordering. Those differences between FTD and FTD-ALS do not survive correction for multiple comparisons.

**Table 2:** Cognitive test performance

Task	Control		bvFTD		FTD-ALS		bvFTD vs control		FTD-ALS vs control		bvFTD vs FTD-ALS	
	n	Median (range)	n	Median (range)	n	Median (range)	z	P value	z	P value	z	P value
Graded naming/30	30	23 (18–29)	23	16 (0–27)	18	11 (1–25)	−4.7	<0.001	−4.9	<0.001	−0.7	0.46
Object name/50	30	50 (49–50)	23	45 (6–50)	11	47 (34–50)	−5.1	<0.001	−4.7	<0.001	−0.7	0.48
Action name/50	30	50 (46–50)	22	47 (6–50)	11	49 (27–50)	−4.8	<0.001	−3.4	0.001	−0.9	0.39
Pyramids palm trees pictures/52	30	51 (49–52)	22	47.5 (20–51)	18	49 (23–52)	−4.9	<0.001	−3.5	0.001	−0.5	0.65
Pyramids palm trees words/52	30	52 (50–52)	22	49 (22–52)	17	49 (21–52)	−5.0	<0.001	−4.3	<0.001	−0.2	0.85
PALPA spelling/40	30	39 (30–40)	22	31.5 (0–40)	14	24 (4–40)	−4.0	<0.001	−3.8	<0.001	−0.8	0.42
PALPA sentence comprehension/24	30	24 (23–24)	22	19.5 (9–24)	13	22 (11–24)	−4.9	<0.001	−4.6	<0.001	−0.7	0.51
Sentence ordering/10	30	10 (7–10)	21	8 (0–10)	16	3.5 (0–10)	−4.8	<0.001	−5.0	<0.001	−1.8	0.07
Letter fluency index*	29	8.7 (5.7–25.3)	20	31.8 (11.6–177)	14	39 (21–195)	−5.4	<0.001	−5.1	<0.001	−1.1	0.29
Category fluency index*	29	3.8 (2.7–6.4)	21	8.9 (3.9–117)	14	21 (6–60)	−5.4	<0.001	−5.3	<0.001	−2.0	0.04
DKEFS block sorting/8	30	5 (2–7)	20	2 (0–6)	12	1.5 (0–6)	−4.8	<0.001	−4.0	<0.001	−0.4	0.66
Brixton errors/54*	30	14 (2–25)	18	16.5 (10–37)	11	26 (10–37)	−2.5	0.01	−3.1	0.02	−1.4	0.17
Hayling connected response time*	30	4 (0–20)	21	17 (0–60)	10	18 (1–60)	−3.8	<0.001	−3.6	<0.001	−0.8	0.42
Hayling unconnected response time*	30	15.5 (0–60)	21	43 (5–60)	10	41 (12–60)	−3.7	<0.001	−3.2	0.001	−0.1	0.90
Hayling inhibition accuracy scaled score/8	30	7 (6–8)	21	1 (1–7)	10	1 (1–6)	−5.4	<0,.001	−4.8	<0.001	−1.0	0.33
Judgement of preference errors	30	0 (0–1)	22	1.5 (0–22)	12	0.5 (0–21)	−3.8	<0.001	−3.7	<0.001	−0.9	0.66
Ekman faces	30	51 (43–57)	22	31 (9–53)	14	26 (14–53)	−5.7	<0.001	−4.4	<0.001	−0.42	0.67

DKEFS: Delis-Kaplan Executive Function System battery

*Higher scores represent greater impairment.

ALS, amyotrophic lateral sclerosis; bvFTD, behavioural variant FTD; FTD, frontotemporal dementia; PALPA, Psycholinguistic Assessment of Language Processing in Aphasia.

### Behaviour

#### Frequency of behaviour change

Behavioural changes were commonly reported in both bvFTD and FTD-ALS and encompassed symptoms within each of the five domains specified by current diagnostic criteria for bvFTD (table 3).^18^ Nevertheless, there were notable differences. Whereas apathy was virtually ubiquitous in both groups, disinhibited behaviours, reduced sympathy and empathy, and repetitive behaviours, particularly simple motor mannerisms, were significantly more common in bvFTD. Changes in dietary habits were also numerically more frequent in bvFTD although differences did not reach statistical significance.

**Table 3:** Frequency of behavioural changes specified in diagnostic criteria

	Percentage showing altered behaviour		χ^2^	P value
	bvFTD n=22	FTD-ALS n=17		
Disinhibition				
Inappropriate behaviour	59	29	3.4	0.07
Loss of manners/decorum	91	59	Fisher’s	0.03^*^
Impulsivity	86	41	8.8	0.003**
Apathy and inertia				
Apathy	96	100	Fisher’s	1.00
Inertia	41	41	0.0	1.0
Loss of sympathy or empathy				
Loss of sympathy	91	53	Fisher’s	0.01^*^
Reduced social interest	77	65	Fisher’s	0.48
Perseverative, stereotyped, ritualistic behaviour				
Simple repetitive	91	47	Fisher’s	0.004**
Complex repetitive	68	29	5.8	0.02^*^
Verbal stereotypies	14	24	Fisher’s	0.68
Hyperorality and dietary change				
Food fads/sweet food preference	86	65	Fisher’s	0.14
Binge eating	55	29	2.5	0.12
Oral exploration of inedible objects	0	0	–	–

*p<0.05; **p<0.01.

ALS, amyotrophic lateral sclerosis; bvFTD, behavioural variant FTD; FTD, frontotemporal dementia.

**Figure 1:**
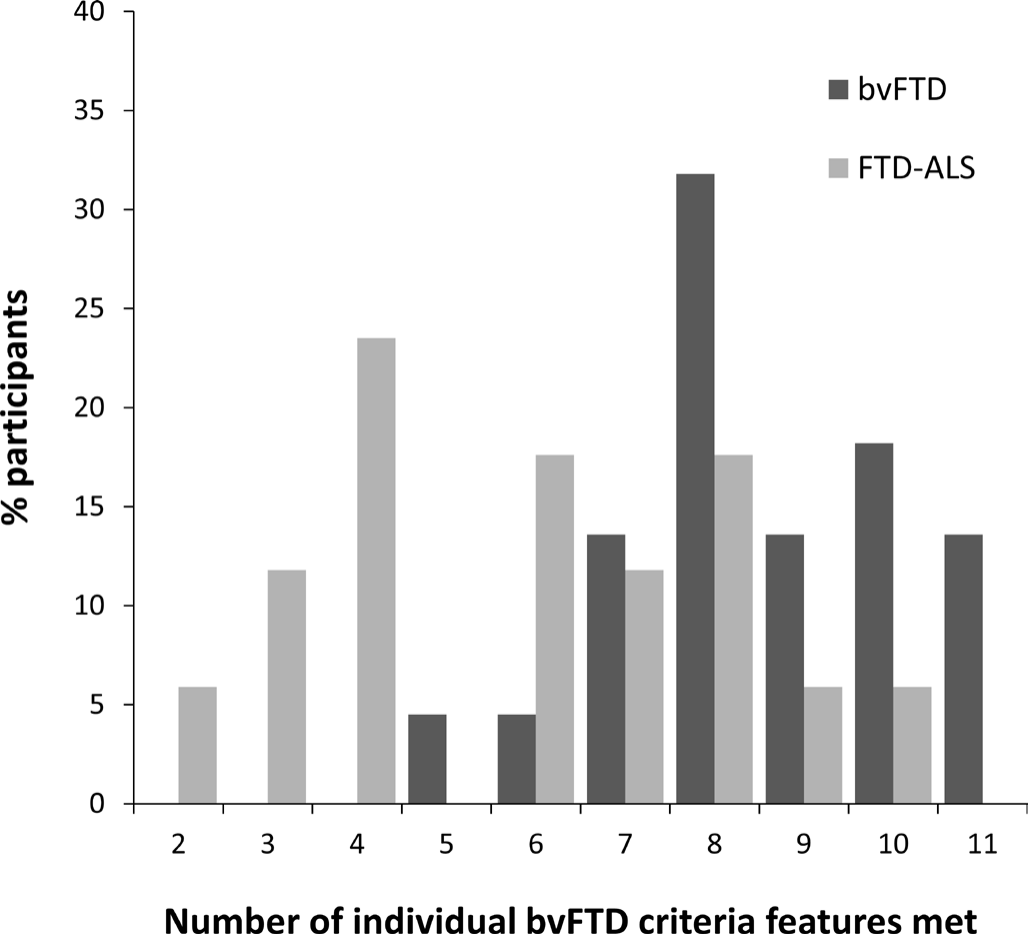
Number of individual features from clinical diagnostic criteria for bvFTD^18^ present in bvFTD and FTD-ALS. frontotemporal dementia; bvFTD, behavioural variant FTD; FTD, frontotemporal dementia.

Overall, informants of patients with bvFTD described in the patient a higher number of altered behaviours than did informants of patients with FTD-ALS (figure 1).

#### Quantitative behavioural scales

The NPI revealed small significant differences between bvFTD and FTD-ALS that were in line with the frequency data: greater agitation and behavioural disinhibition in bvFTD (table 4). There was also a trend towards greater apathy, elation and irritability in bvFTD and depression in FTD-ALS.

**Table 4:** Quantitative measures of behaviour change in bvFTD and FTD-ALS

	bvFTD (n=22)	FTD-ALS (n=17)	Mann-Whitney	
	Median (range)	Median (range)	z	P value
NPI				
Delusions	0 (0–8)	0 (0–10)	−0.0	0.97
Hallucinations	0 (0–8)	0 (0–4)	−0.7	0.48
Agitation	3 (0–8)	0 (0–3)	−2.7	0.01*
Depression	0 (0–6)	0 (0–6)	−1.9	0.06
Anxiety	0 (0–8)	1 (0–4)	−0.2	0.87
Elation	0 (0–8)	0 (0–6)	−1.9	0.06
Apathy	8 (0–12)	4 (0–8)	−2.0	0.05
Disinhibition	3 (0–8)	0 (0–6)*	−2.3	0.02*
Irritability	1 (0–12)	0 (0–2)	−2.0	0.05
Aberrant motor behaviour	1 (0–12)	0 (0–12)	−0.7	0.47
Sleep	0 (0–12)	0 (0–12)*	−0.7	0.91
Appetite	8 (0–12)	6 (0–12)	−1.2	0.25
FrSBe				
Apathy change	36.5 (5–52)	29 (7–49)	−0.73	0.46
Disinhibition change	17.5 (3–57)	10 (−4–32)	−2.19	0.03*
Executive change	34 (3–64)	18 (8–47)	−2.13	0.03*

*n=16; *p<0.05.

ALS, amyotrophic lateral sclerosis; bvFTD, behavioural variant FTD; FrSBe, Frontal Systems Behaviour Scale; FTD, frontotemporal dementia; NPI, neuropsychiatric inventory.

The FrSBe elicited greater change in bvFTD in the disinhibition and executive but not apathy domains of behaviour (table 4). The level of apathy, disinhibition and executive dysfunction reported by informants before the onset of the patient’s illness did not differ in the two groups, excluding the possibility that differences were influenced by premorbid factors.

#### Self-report versus informant-based report

The FrSBe data are based on informant-based reports of behavioural change. Self-reports were also obtained from a subsection of patients (15 bvFTD and 8 FTD-ALS). Patients in both groups reported less change than their corresponding informant, although the disparity between self and informant report was greater and reached statistical significance only in bvFTD (table 5).

**Table 5:** Self versus informant-based report of behaviour change in bvFTD and FTD-ALS

Group	FrSBe	Self-report	Informant report	Wilcoxon	
	Domain	Median (range)	Median (range)	Z	P value
bvFTD	Apathy	1 (−2–29)	38 (5–52)	−3.4	0.001**
	Disinhibition	0 (−8–31)	19 (3–57)	−3.4	0.001**
	Executive	8 (−2–29)	41 (3–64)	−3.4	0.001**
FTD-ALS	Apathy	9 (0–27)	26.5 (9–34)	−1.7	0.09
	Disinhibition	3.5 (−1–14)	13 (−2–17)	−1.9	0.06
	Executive	8 (0–24)	14 (8–38)	−1.3	0.21

**p<0.01.

ALS, amyotrophic lateral sclerosis; bvFTD, behavioural variant FTD; FrSBe, Frontal Systems Behaviour Scale; FTD, frontotemporal dementia.

### FTD-ALS relationships

#### Cognitive versus motor onset

No significant cognitive or behavioural differences were identified in patients with FTD-ALS depending on whether cognitive or motor symptoms were noticed first.

#### Cognitive clinic versus Motor Neurone Disease (MND) clinic

Patients presenting to a cognitive clinic showed greater disinhibition than those presenting to an MND clinic (z=−2.3, p=0.02). Other comparisons were non-significant.

#### Bulbar signs

Patients with FTD-ALS with bulbar signs exhibited greater cognitive impairment than those without, particularly on language tasks: object naming z=−2.3, p=0.02, action naming z=−1.9, p=0.05, PALPA spelling z=−2.6, p=0.01, PALPA sentence comprehension z=−2.3, p=0.02, sentence ordering z=−2.5, p=0.01, DKEFS block sorting z=−2.1, judgement of preference z=−2.2, p=0.03. No behavioural differences were apparent. Patients with bulbar signs were younger (mean 62 years) than those without (mean 70 years) t=−2.4, p=0.03. They did not differ in symptom duration.

### Genetics

Thirty-three patients (19 bvFTD and 14 FTD-ALS) were screened for the *C9ORF72* hexanucleotide repeat expansion and 27 for other known FTD genes (17 bvFTD and 10 FTD-ALS). Six bvFTD (32%) and seven FTD-ALS (50%) patients were positive for the *C9ORF72* expansion. Two patients with bvFTD had a mutation in the *MAPT* gene and two in the progranulin gene.

Patients with the *C9ORF72* expansion performed more poorly than those without on spelling, sentence comprehension and block sorting and there was a trend towards poorer sentence ordering, semantic association and category fluency (table 6). No significant differences were elicited on behavioural measures or severity of illness measured by duration of illness or CDR ratings.

**Table 6:** Cognitive test performance as a function of *C9ORF72*

Task	C9 +ve		C9 –ve		Mann-Whitney	
	n	Median (range)	n	Median (range)	z	P value
Graded naming/30	13	13 (7–25)	20	17 (0–27)	−0.70	0.48
Object name/50	9	45 (34–50)	19	47 (6–50)	−0.92	0.36
Action name/50	9	47 (27–50)	19	48 (6–50)	−0.57	0.57
Pyramids palm trees pictures/52	13	45 (23–52)	19	49 (20–52)	−1.75	0.08
Pyramids palm trees words/52	13	47(21-52)	19	49 (22–52)	−0.85	0.40
PALPA spelling/40	10	21 (4–35)	17	33 (5–40)	−2.19	0.03*
PALPA sentence comprehension/24	9	17 (12–22)	19	23 (9–24)	−2.09	0.04*
Sentence ordering/10	11	5 (0–9)	18	8 (0–10)	−1.86	0.06
Letter fluency index	10	50 (19–177)	15	30 (12–103)	−1.51	0.13
Category fluency index	11	22 (6–117)	17	9 (14–116)	−1.90	0.06
DKEFS block sorting/8	9	1 (0–2)	17	3 (0–6)	−2.01	0.05

DFEFS: Delis-Kaplan Executive Function System battery

*p<0.05.

PALPA, Psycholinguistic Assessment of Language Processing in Aphasia.

## Discussion

This prospective study examined the hypothesis, arising from our earlier retrospective study,^16^ that bvFTD is associated with greater behavioural change and FTD-ALS more marked language change. The current study, involving an independent cohort of patients, provided a more in-depth analysis of behaviour, language, executive and social cognition than previously available. It incorporated measurements of severity as well as presence/absence of abnormality, test procedures that control for motor deficits, the inclusion of a healthy control group and comparisons of behavioural change based on patients’ and informants’ report. It explored the relationship of cognitive/behavioural change to motor disability in FTD-ALS and the influence of genetic mutations. The study’s prospective nature confers the advantage of more systematic and controlled administration of cognitive tests and behavioural interviews by a single examiner.

The bvFTD and FTD-ALS groups both showed striking impairments in cognitive performance compared with controls, confirming the sensitivity of the language, executive and social cognition measures to FTD. The two patient groups showed largely similar cognitive profiles. The data did, however, suggest subtle differences: poorer performance in FTD-ALS in category fluency and a trend towards poorer performance in ordering words to form a grammatical sentence. Those differences need to be interpreted with caution, because they do not survive correction for multiple comparisons. Nevertheless, the differences are not arbitrary. Poor verbal fluency has been documented as a prominent feature of ALS.^30^ and has been identified as poorer in FTD-ALS than bvFTD using different fluency measures. The greater difficulty cannot be ascribed to motor slowing in FTD-ALS because the fluency measures control for motor speed by calculating the time to generate items in relation to the time to read/copy those same items. The suggestion of greater problems in grammar in FTD-ALS than bvFTD is in keeping with findings from our previous retrospective study involving an independent cohort of patients.^16^ They are consistent too with independent reports of syntactic impairments in both FTD-ALS and ALS.^33 34^ Studies have also identified significant semantic impairments in FTD-ALS.^35^

Behavioural changes were common in both patient groups (in keeping with the selection criteria that patients should fulfil criteria for the behavioural form of FTD on the basis of behaviour and executive changes). Nevertheless, whereas apathy predominated and was ubiquitous in FTD-ALS, patients with bvFTD showed more widespread behavioural changes, andtypically endorsed more behavioural features from diagnostic criteria. Disinhibition, impulsivity, loss of empathy and repetitive behaviours were all significantly more common in bvFTD. These findings reinforce previous observations.^14 16^

Severity of illness is unlikely to provide an adequate account of observed behavioural differences. Despite differences in duration of symptoms, severity as measured by the FTLD modified CDR^17^ did not differ between groups. Moreover, the two groups were largely matched in terms of their cognitive performance and where differences occurred these were in the direction of poorer performance in FTD-ALS. Furthermore, if behavioural differences were an artefact of disease severity alone more behavioural changes overall might be anticipated but not differential impairment in specific domains. Arguably, the physical limitations in people with FTD-ALS might reduce patients’ capacity to exhibit certain behaviours, such as repetitive behaviours or disinhibition. It is also possible that caregivers might under-report behaviour changes. Their focus on practical management of patients’ physical disability might reduce their attention to behaviour, or else they might attribute behavioural changes to a natural reaction to a life-changing diagnosis. Yet, caregiver under-reporting would not account for the disproportionately high occurrence in FTD-ALS of apathy relative to other domains of behavioural change. The semistructured interview techniques, with provision of specific examples of behaviour and the comparison of behaviours before and after illness onset, aimed to mitigate potential secondary effects of ALS. Patients with bvFTD significantly under-reported behaviour changes compared with their informant. The disparity between informant and self-report of symptoms was, moreover, substantially greater than in the FTD-ALS group. This novel finding suggests a greater reduction in insight in bvFTD, in keeping with their more marked behavioural change.

Within the FTD-ALS cohort, there were no cognitive or behavioural differences as a function of nature of onset: cognitive/behavioural vs motor. While small numbers might arguably explain the lack of statistical difference comparably small numbers did elicit systematic statistical differences on language tasks in patients with and without bulbar signs. The findings suggest that onset type is not a major determining factor and the terms FTD-ALS and ALS-FTD may be used interchangeably. It is instructive that patients with FTD-ALS presented more commonly to an ALS than specialist dementia clinic, yet more caregivers noted behavioural/cognitive before motor symptoms. Moreover, some caregivers reported simultaneous development of motor/cognitive symptoms or expressed uncertainty, suggesting that the evolution of symptoms may be blurred. Findings from other studies suggest that the designation FTD-ALS may be more appropriate than ALS-FTD. A large study of ALS^11^ distinguished between motor and behavioural-predominant phenotypes. Although patients with motor presentation developed alterations in cognition and behaviour over time these were less severe and more circumscribed than in patients with behavioural presentation and did not fulfil criteria for bvFTD. In ALS-FTD, motor symptoms rarely preceded the onset of behaviour change. Other authors have highlighted the lack of congruity between motor and cognitive/behavioural decline in ALS,^14^ again suggesting that the term FTD-ALS might be a more appropriate designation for the behavioural disorder.

The potential influence of genetic factors is intriguing. Repeat expansions in the *C9ORF72* gene were present in six bvFTD and seven patients with FTD-ALS: 32% and 50%, respectively of those who were tested. Tasks on which expansion carriers were more impaired than non-carriers, or showed a trend towards greater impairment, all make substantial linguistic demands: spelling, sentence comprehension, block sorting based on semantic/verbal rules, sentence ordering, semantic association and category fluency. Such a pattern suggests a specific association between language system dysfunction and *C9ORF72* repeat expansions. This might feasibly drive the subtly greater language impairments in FTD-ALS than bvFTD observed in this study.

We did not observe neuropsychiatric differences in people with and without the *C9ORF72* expansion contrary to previous reports.^8 9 36^ This likely reflects the small numbers and, possibly, selection bias against psychotic symptoms in a study requiring voluntary participation.

The small number of patients with *C9ORF72* expansions precluded meaningful sub-comparisons of cognition and behaviour in bvFTD and FTD-ALS as a function of *C9ORF72*. Nevertheless, the findings in the whole *C9ORF72* positive group are sufficient to suggest that the repeat expansion exerts an influence on patients’ cognitive profile.

*C9ORF72* repeat expansions might also feasibly contribute to group differences in the proportion of patients showing frontotemporal atrophy on structural neuroimaging. A normal scan or generalised atrophy occurred in both groups in association with the presence of *C9ORF72* expansions. This is in line with previous observations that atrophy in *C9ORF72* patients may be less strikingly focal than in other forms of FTD.^8 9^ Genetic screening was not available for one bvFTD and five patients with FTD-ALS in whom generalised atrophy was reported. The possibility of *C9ORF72* positivity in those patients cannot be excluded. Clinical scans were carried out in different diagnostic centres so reporting differences can also not be ruled out.

The principal limitation of the study is the relatively small size of the bvFTD and FTD-ALS groups. Some participants could not complete all tasks, further diminishing group size. There was, in consequence, inherently limited statistical power to detect differences, particularly as the cohort of patients proved to be variable with regard to severity of symptoms, despite endeavours to select patients in the mild-to-moderate stages of disease. The data do, nevertheless, reinforce findings from our earlier retrospective study^16^ involving an independent patient cohort and they serve as pointers to possible differences that require prospective investigation in larger-scale studies.

Within-group heterogeneity was particularly evident on cognitive testing, with some patients in both groups showing impairment on language tasks and others performing relatively well. The suggestion in this study that genetic factors may play a role highlights also the need to consider distinct genetic and sporadic variants in future large-scale comparative studies of bvFTD and FTD-ALS.

A related limitation of this study stems from the fact that a large battery of tests was administered. The rationale was to encompass the spectrum of cognitive and behavioural domains affected in bvFTD. The inevitable consequence is that the relatively subtle group differences do not survive correction for multiple comparisons. As noted above, however, identified differences were not isolated but rather constitute a coherent pattern, and are in line with predictions and previous findings. They are unlikely therefore to have occurred due to chance alone.

The possibility cannot be excluded that some patients in the bvFTD group will later develop ALS. Indeed, two patients initially recruited into the bvFTD group were later reclassified. However, misclassifications are likely to be rare. Of the 23 patients with bvFTD in the study, 21 were followed up for at least 1-year post-study and had not exhibited signs of ALS. Moreover, the mean duration of symptoms in the bvFTD group at the time of assessment was 5 years. Current evidence suggests that the risk of developing ALS declines with the duration of FTD symptoms and is unlikely after 5 years.^37^ In any event, misclassifications would have the effect of masking rather than exaggerating differences between bvFTD and FTD-ALS, suggesting that identified differences are likely to be real.

There are potential clinical implications of the study. If prominent verbal fluency and other language difficulties, occurring in the context of prevailing apathy, prove to be predictors of FTD-ALS, then patients with bvFTD exhibiting those symptoms might be especially vulnerable to developing ALS and should be monitored closely.

There are potential theoretical implications too. The notion of a continuum of disease between FTD and ALS^3 10^ is attractive, yet it presents challenges. Heterogeneity in underlying pathology and genetic mutations suggests that not all patients with FTD are vulnerable to developing FTD-ALS. Our findings indicate commonalities between bvFTD and FTD-ALS but also sufficient differences to raise the possibility of FTD-ALS as a distinct clinical phenotype. We speculate that FTD-ALS is not simply the summation of ALS and FTD, but rather a specific behavioural/cognitive entity, allied to bvFTD but with specific pathology, linked genes and clinical characteristics.

## Conclusion

The data suggest subtle differences between bvFTD and FTD-ALS in both behavioural and language profiles, which are not simply a function of illness duration or overall severity of disease. Repeat expansions in the *C9ORF72* gene may contribute to those differences. A task of future studies is to clarify the factors that contribute to phenotypic variation, both within and between these groups.
